# It All Adds Up over Time: Cumulative Lead Exposure and Cognition in Older Women

**DOI:** 10.1289/ehp.117-a162b

**Published:** 2009-04

**Authors:** David J. Tenenbaum

Many older people in the U.S. population were chronically exposed to lead from paint and gasoline prior to the 1980s. To date, most of the research on lead and cognitive functioning in older age has focused on men, despite the fact that women live longer on average and therefore may be more likely to develop dementia over the course of their life span. Now, in a prospective look at a subset of data from the Nurses’ Health Study—which began in 1976 and included 121,700 registered nurses aged 30–55 years—researchers report that even low-level cumulative lead exposure may exacerbate cognitive decline in older women **[*EHP*** **117:574–580; Weuve et al.]**.

The study looked at 587 women (now aged 47–74 years) who had undergone bone lead evaluations as part of two studies during the 1990s; to assess long-term exposures, bone lead concentrations were determined at each woman’s mid-tibial shaft (shin bone) and patella (kneecap). All but 6 of those individuals had also provided blood samples for assessment of more recent lead exposure.

Trained interviewers conducted telephone interviews an average of 5 years after the lead measurements were taken to obtain cognitive data. The interviewers asked participants to perform a variety of tasks related to memory and verbal abilities.

The researchers found a significant positive association between cognitive deficits and higher lead levels in the tibia but not in the patella or blood. Because the type of bone in the tibia is known to provide a longer record of lead exposure than other tissues, the research points to long-term exposure to lead—but not to current or recent exposures—as the most likely source of deterioration in cognitive functioning in this population. One standard deviation increase in lead exposure produced, on average, as much decrement in cognitive functioning as 3 years of aging in the women in the study.

Lead may damage brain neurons through a range of mechanisms, including oxidative damage and programmed cell death. As the population of older adults grows, it becomes ever more critical to understand ways to ward off dementia. Clues to this understanding may come from studying subtle decreases in cognitive functioning, which, as several researchers have found, often precedes the development of dementia. If other studies confirm the observed relationship between cumulative lead exposure and impaired cognition, measures to minimize exposure or reduce the body’s lead burden could have a substantial impact on aging-related cognitive impairment.

